# The Hospital

**Published:** 1887-02-05

**Authors:** 


The Hospital
First Special Supplement.

### A Revolution in Hospital Management.*

### THE NEWCASTLE INFIRMARY.

### SUGGESTIVE REPORT OF THE SPECIAL COMMITTEE.

The Special Committee appointed by the Governors of Newcastle Infirmary two months ago, to inquire into the management and financial position of the institution, have completed their labours, and have adopted the following report for presentation to the Governors, which we commend to the attention of our readers, in conjunction with the leading article published on page 293 of last week's Hospital.

The Special Committee appointed to confer with the House Committee as to the past management of the Newcastle Infirmary, and the best mode of extricating the institution from its present financial position, and to report to a special Court of Governors what they recommend should be done under the circumstances, have held thirteen conferences with the House Committee and the medical staff, and have obtained evidence from various sources. It was resolved to commence the inquiry by considering whether the work of the hospital is conducted economically, so far as is consistent with efficiency.

#### ADMISSION OF PATIENTS.

The Committee first inquired into the method adopted in the admission of patients, with a view of learning whether persons ineligible for the charity were allowed to enjoy its benefits. Last year, there were in the Infirmary on January 1st, 236 patients, 2,846 persons were admitted during the year, of whom 1,294 were admitted by letter; 722 as emergency cases; and 112 as Magdalen cases; and 718 as accidents.

The emergency cases consist of persons who come to the Infirmary suffering from disease which requires immediate attention, and are admitted by the resident medical staff without letters. The committee were informed that, upon the whole, those admitted as emergency cases are less able to pay than those admitted by letter; and that even among the letter patients there are not a large proportion who could afford to pay the total cost of treatment, though doubtless there are some who could contribute a small weekly sum towards the expenses of their maintenance, *e.g.*—It occasionally happens that men who have no families to support, receive sick pay from their society all the time they are maintained by the public in the Infirmary, and such persons make no contribution to the funds of the institution. The committee, therefore, suggest that the governing body should consider whether it would not be possible to set apart a ward for the reception of patients who are in a position to pay the whole or part of the cost of their maintenance in the institution.

#### MAGDALEN WARD.

The expenses incurred in connection with the inmates of the Magdalen Ward are less in proportion than that of the other patients in the house, because their services are called into requisition in scrubbing and other household work; but the committee are of opinion that the burden of their maintenance should not be entirely thrown on this institution, and that it is a question whether the Guardians might not be expected to contribute a portion of the expenditure.

#### CHILDREN'S WARD.

There are thirty beds in this ward; these are always full, and there are generally about ten children in other wards. Your committee are of opinion that when the new Children's Hospital in Newcastle is completed, it would conduce to the welfare of both institutions if the Infirmary were to refuse to admit children, with the possible exception of certain surgical cases.

At present there is much overlapping among the medical institutions of the district. Expense is thereby increased, and no corresponding advantage gained. If the Infirmary continues to receive children we shall have, ere long, three practically rival institutions—viz., the Children's Ward of the Infirmary, the Newcastle Children's Hospital, and the Gateshead Children's Hospital.

The removal of the children from the Infirmary would lighten the pressure on that institution, and would inflict no hardship on the children, as there will be adequate accommodation for them in the hospitals specially erected for them.

#### ADMISSION REGISTERS.

The Committee recommend that greater care should be taken in the filling up of the books of admission, and that the medical officer admitting the patient should attach his name or initials to the entry in all accident and emergency cases. They further recommend that columns be provided in the admission book for the name of the employer. And that, on application to the House Governor, officials of trade and friendly societies be permitted to examine the admission books.

* Special Prizes of Ten Guineas and Five Guineas, will be given for the two best criticisms of the points dealt with by this report, accompanied by Tables giving the details as to the cost of Food and Stimulants similar to those given on page 2 of this report, and an equally detailed statement as to the expenditure on (1) Drugs and Surgical Appliances, (2) Domestic Expenses, (3) Salaries and Wages, and (4) Incidental Expenses for the year 1886, of the particular hospital with which each competitor may be connected. Full particulars of the conditions and form of contributions will be found on page 318 of The Hospital for February 5th, 1887.

REPORT OF THE SUB-COMMITTEE ON FOOD AND STIMULANTS.

The sub-committee appointed to report upon the expenditure of food at the Newcastle Infirmary have, by the kindness of the secretaries and officials of several medical institutions, been furnished with particulars which enabled them to draw up the following table of the comparative expenditure on food and stimulants at seven different hospitals. In comparing the relative expenditure of these several institutions, it is important to bear in mind that surgical cases require a much more liberal diet than medical patients, and, therefore, an institution like the Newcastle Infirmary, where there were no less than 1,129 operations last year, must cost much more per occupied bed than an institution like Sunderland, where there were only 360 operations. Also, the more severe the class of operations the greater the need of costly forms of nourishment; and, therefore, for a perfectly fair comparison, it is necessary to obtain statistics of the number and severity of the operations. These your committee found it hard to procure. They give, however, the following tables. which they have compiled with some care :

*Hospitals and Infirmaries*, 1885—*Analysis of Costs per Occupied Bed* (*Food and Stimulants*).

| | Newcastle. | Sunderland. | Leeds. | Sheffield. | Birmingham General. | Edinburgh. | Glasgow Western. | Dundee. |
|---|---|---|---|---|---|---|---|---|
| Meat:— | £ s d | £ s d | £ s d | £ s d | £ s. d. | £ s. d. | £ s. d. | £ s. d. |
| (a) Beef and mutton | 10 3 2½ | 6 7 5¾ | 7 7 0 | 8 1 4½ | 7 13 8¾ | 7 9 10¾ | 6 17 6 | 5 9 4 |
| (b) Bacon and ham .. | 0 9 9 | 0 4 8¼ | 0 10 1¼ | | 0 8 1 | 0 9 5¼ | 0 3 0 | 0 2 10 |
| Fish ... } Poultry and Game .. } | 1 8 5¼ | | 1 2 3¼ | 2 6 4½ | 1 17 0¼ | | | |
| Fish | | 0 4 7 | | | | 0 10 11 | 0 9 8½ | 0 12 8 |
| Poultry and Game | | 0 2 10 | | | | 1 3 2¾ | 1 9 0 | 0 3 9½ |
| Bread and Flour ...... | 2 6 2 | 2 2 0 | 1 8 2¾ | 1 11 5 | 1 8 3¾ | 1 17 3½ | 1 18 6 | 1 17 3 |
| Oatmeal ... | 0 0 3½ | — | 0 1 3 | | 0 0 11½ | 0 6 4½ | 0 10 5½ | 0 2 0 |
| Barley ... | 0 0 6¼ | 0 0 0½ | 0 0 6½ | | 0 0 1 | 0 1 7 | 0 0 9¼ | 0 4 6½ |
| Cheese ... | 0 3 4 | 0 1 7½ | 0 2 6¾ | | 0 3 0½ | 0 2 1¼ | 0 0 10¾ | 0 1 0 |
| Greengroceries ... | 0 15 6 | 0 9 10½ | 0 15 7¼ | 0 15 2½ | 0 12 4¼ | 0 8 6 | 0 8 6 | 0 6 0 |
| Milk ... | 4 14 3 | 3 1 4½ | 3 6 1 | 2 8 6½ | 3 2 6 | 3 18 9 | 3 10 1¾ | 2 3 1¼ |
| Tea ... | 0 9 9½ | 0 14 8 | 0 15 1½ | | 0 7 10½ | 0 14 8 | 0 16 6 | 0 15 6 |
| Butter ... | 0 14 9½ | 2 0 3¼ | 2 3 7½ | | 0 16 11¼ | 1 10 8½ | 1 14 1 | 0 18 9 |
| Coffee ... } Cocoa ... } | | | 0 2 8 | | 0 2 6 | 0 4 4¼ | | |
| Coffee | 0 1 10 | 0 2 5 | | | | | 0 3 11 | 0 1 6 |
| Cocoa | — | 0 0 5¾ | | | | | — | — |
| Sugar ... | 0 5 5¼ | 0 8 9½ | 0 10 8¼ | | 0 5 1¾ | 0 7 8 | 0 11 3¼ | 0 9 2½ |
| Rice ... } Other farinaceous food } | | | 0 1 9 | | 0 3 2¼ | 0 4 9 | | |
| Rice | 0 2 0 | 0 1 4¼ | | | | | 0 1 4 | |
| Other farinaceous food | 0 0 5¼ | 0 0 2¾ | | | | | 0 2 5 | |
| Eggs ... | 0 7 8¾ | 0 8 2¾ | 0 6 6¾ | | 0 12 5¼ | 0 17 11 | 1 0 0½ | 0 7 3½ |
| Items not included in above ... | 1 0 4¼ | 0 19 2 | 0 19 4 | | 0 7 5 | 0 0 10½ | 0 10 10½ | 0 2 6 |
| Total cost of food ...... | 23 3 10 | 17 10 1 | 19 13 5[illegible] | 29 12 4 | 18 1 7 | 20 9 0 | 20 18 11 | 13 17 3 |
| Wine ... | 0 4 4½ | 0 1 3½ | 0 2 9½ | | 0 3 7¼ | 0 3 4¾ | 0 5 4 | 0 6 7½ |
| Beer ... | 0 7 9 | 0 1 2¼ | 0 3 10¾ | 0 15 8 | 0 12 4¼ | 0 5 2¾ | 0 6 0 | 0 0 3 |
| Spirits ... | 0 15 3½ | 0 2 0¾ | 0 2 8¾ | | 0 6 7½ | 0 9 6½ | 0 9 0 | 0 7 3½ |
| Total cost stimulants per occupied bed .... | 1 7 5 | 0 4 6¾ | 0 9 5 | 1 18 10 | 1 2 7 | 0 18 0 | 1 0 4 | 0 14 2 |
| Number of patients in the year ... | 3082 | 1444 | 4401 | 1812 | 3545 | 7854 | 3215 | 2098 |
| Average stay in hospital (days) ... | 30·1 | 32 | 21 | 32 | 29 | 27·6 | 41·8 | 27·14 |
| Number of persons other than patients whose food is included in above totals ...... | 85 | 44 | 130 | 54 | 106 | 225 | 122 | 55 |

All the cost figures in these tables are obtained by multiplying the number of patients by the average stay in the hospital, divided by the number of days in the year, and dividing the sum paid for each item supplied to the institute by the result.

*Major operations in various Hospitals compared*, 1885 (*but only those performed by Honorary Staff*).

| Nature of operation. | Newcastle. | Sunderland. | Leeds. | Sheffield. | Birmingham General. | Edinburgh. | Glasgow. Western. | Dundee. |
|---|---|---|---|---|---|---|---|---|
| Major amputations ... | 51 | 16 | 61 | 14 | 36 | — | 61 | 15 |
| ,, joint excisions ... | 32 | 4 | 55 | 5 | 8 | — | 25 | 9 |
| ,, breast ,, ... | 22 | 12 | — | 1 | 18 | — | — | — |
| Ovariotomies ... | 13 | 5 | 19 | — | 22 | — | 9 | 1 |
| Herniotomies ... | 7 | 2 | 20 | 11 | 18 | — | 18 | 6 |
| Abdominal section (not in above) | 5 | — | 2 | — | 6 | — | — | — |
| Other major operations ... | 45 | 6 | 63 | 17 | 37 | — | 24 | 36 |
| Totals ... | 175 | 45 | 220 | 48 | 145 | — | 137 | 67 |
| Total operations of all kinds .... | 1129 | 360 | 1585 | 531 | 365* | — | 794 | 213 |

It will be seen, from reference to the table given, that the expenditure for beef and mutton at the Newcastle Infirmary is largely in excess of that of any other institution. Your committee have endeavoured to ascertain whether there was any considerable waste in connection with the kitchen arrangements, or in the distribution of the food in the wards, and they are of the opinion that the methods of contracting for the supplies, checking the quantities, cooking and distributing the food, are fairly satisfactory. and that little waste occurs. The amount supplied depends on the orders given by the medical staff, and is much or little, according to the dietary prescribed by them. We desire to call the attention of the medical staff to the very large expenditure on beef tea, which last year amounted to £747. This, to a very great extent, accounts for the excessive expenditure on meat at the Newcastle Infirmary. We also desire to draw the attention of the medical staff to the amount of beef used in the preparation of beef tea, which is 1 lb. to the pint. We are of opinion that 10 oz. to the pint will make excellent beef tea for all ordinary purposes, if it be cooked at a lower temperature, and for a longer time. The committee would also ask the Medical Board to consider whether, in some cases, skimmed milk could not be substituted for beef tea as a more economical means of supplying the required nutriment.

We also think that better special beef tea than that now employed for enemata could be obtained from 1lb of meat to the pint, if the temperature at which it is cooked be not allowed to exceed 150 deg. Fahrenheit.

The committee venture to suggest to the Medical Board the expediency of giving porridge instead of bread for breakfast; for, if this was done, the patients obtaining more proteid matter than they now do at breakfast, the allowance of meat at dinner might be reduced 1oz., and the patients not be losers thereby. The expenditure of £1 8s. 5½d. per occupied bed on fish and poultry points to a somewhat lavish ordering of extra diets. Evidence which was adduced has convinced the committee that the method of ordering the extra diets has been somewhat lax; and they recommend that a more clearly understood method of marking the dietary on the patients' cards be adopted, that the medical staff be more careful in marking the diets, and that the nurses be instructed as to the meanings of the signs used. We are also of the opinion that the entries on the cards should be compared more frequently with the entries in the diet book, and no diet should be sent upstairs unless entered in the diet book.

Considering the large expenditure upon milk, the committee would suggest whether, for many purposes, skimmed milk could not be used, as is done in other hospitals. The committee believe that a larger amount of nutritive matter would be extracted from the materials used in making soup if a slower method of cooking, at a lower temperature, were adopted.

In conclusion, the committee are of opinion that, by careful revision of the dietary, and tables by the Medical Board, and the substitution of more economical forms of nourishment, *e.g.*, rabbits instead of chicken, the expenditure on food ought to be reduced below that of Leeds, where in addition to the patients, 130 persons are supplied with food, as against 85 at the Newcastle Infirmary, a reduction of about £4 per bed, which would be equivalent to a saving of £938 per annum. Indeed, we see no reason why our expenditure should not be reduced even more than this till it falls to the scale of the Birmingham General Hospital.

STIMULANTS.

The expenditure on stimulants is considerably in excess of the amount used at any of the other institutions with which we have instituted a comparison, with one exception, and ought to be capable of a reduction of at least 10s. per bed. This would be equivalent to a saving of £117 on this head.

GENERAL RECOMMENDATION.

As has been pointed out above, the amount of the expenditure depends on the action of the medical staff; and, as each member of that staff acts independently, there are at present no means of ascertaining who is responsible for any increase of expenditure. The committee, therefore, recommend that the plan in vogue at Edinburgh and some other hospitals should be adopted, and an accurate account be kept of the amount expended by the order of each member of the medical staff. Such account should include drugs, dressings, and surgical instruments, as well as food and stimulants.

It should be borne in mind that a saving of 1d. per day per patient means a reduction of £400 per annum in the expenditure of the institution.

W. Moore Ede.
J. H. Rutherford.
H. E. Armstrong.

The recommendations of the committee with regard to beef-tea are, to a great extent, borne out by the very interesting analysis of a sample of beef-tea sent to Dr. Walker, Analyst for the city of Carlisle, which is of such public interest that we give it in full:—

*Analysis of a Sample of Beef Tea received from the Newcastle Infirmary, and Report thereon, December 13th, 1886.*

| | |
|---|---|
| Saline matter (equal to ·13oz. or 62 grains per pint) | ·67 per cent. |
| Gelatine and extractive (dried for four hours in vacuo), including ash (equal to 47 oz. or 225 grains per pint, after deducting ash or salts)...... | 2·9 per cent. |
| Albumen and Hœmatin (equal ·08 oz. or 38 grains per pint. Peptone obtained from this by dissolving in pepsin, and precipitating 36 grains per pint ........ | ·39 per cent. |
| Total peptone and extractive obtained by digesting the beef-tea with acid and pepsin, and evaporating (including salts). (Equal ·67 oz. or 321 grains per pint. Total peptone obtained by digesting the tea with pepsin, and precipitating ·086 or 41 grains per pint........ | 3·4 per cent. |

I was not informed what quantity of beef was used in making the beef tea, but, from the analysis, I should think 1lb. has been used for each pint. The extract and salts indicate that rather more than a lb. has been used, but this excess may be from the tea having been concentrated by evaporation. If I am correct in assuming that each pint represents 1lb. of beef, the amount of albumen and hœmatin is very much below what it should be, as it is only equal to 2 or 3 oz. of beef. I extracted from a sample made by myself, with the same beef, 228 grains, instead of 38, *i.e.*, about six times the amount found in the beef tea. This deficiency of albumen is of importance, as the whole of the nutritive value of the tea depends on the albumen, the extract, consisting of flavouring matters, salts, and gelatine, experiment has proved (what we should expect) that it is only a stimulant, and has little or no nutritive value.

The whole of the albumen was in an insoluble form, but was readily dissolved by pepsin, giving 22 grains of peptone. This is rather below the amount of peptone obtained by digesting the beef tea with pepsin, and precipitating; but, doubtless, in this latter case, some other matters are carried down with the peptone, but even taking this latter quantity as a standard, the tea has only a nutritive value of 41 grains, instead of 260 or 280 grains.

I have not had time to make any further experiments, but I think it probable that even this amount of albumen might be exceeded; but, certainly, by employing a more scientific method, a tea might be made having a nutritive value four to six times greater than the sample sent for analysis.

As the whole of the albumen is precipitated by boiling in making the tea, the beef tea should not be used for enemata. When the beef tea is intended for this purpose, it should not be heated above 100 deg. Fahrenheit.

The extract tested by Liebig's process was found to be of very good quality, and, in every other respect, apart from the deficiency in albumen, the beef tea was of excellent quality.

I shall be glad to give any further information, or make further experiments to devise a method by which the greatest amount of albumen, etc., can be retained.

I am, your obedient servant,
T. Hatfield Walker, F.C.S,
Analyst for the City of Carlisle.

CASES OF DRUNKENNESS AND VIOLENCE.

As reports have been circulated to the effect that a large number of patients are brought to the Infirmary in consequence of injuries received while in a state of intoxication, the committee endeavoured to ascertain how many such cases had been admitted during the previous year; but finding that no accurate record had been kept, the committee requested that, for the future, an account be kept showing, as far as practicable, the number of cases of patients admitted and treated in the Infirmary, which can with reason be considered as primarily chargeable upon authorities responsible for the public peace and good order of the city—particularising cases arising from drunkenness and violence.

From the record which was kept at the request of the committee, it appears that between November 18th, 1886, and January 10th, 1887, there were 47 cases of persons brought to the Infirmary in a state of intoxication, who, while in that condition, had suffered bodily injury of more or less severe character. This does not include those who may have been injured when in a state of intoxication, but who, when they come to have their injuries treated, were sober. There were, in the same period, 91 cases of injury due to deliberate acts of violence. The number was probably in excess of the above, as many when they come for treatment allege as the reason of their accident to have been a "fall," an expression which covers a variety of cases.

The committee then proceeded to consider the expenditure of the establishment, and obtained some very valuable evidence from the secretary of the Sunderland Infirmary which led them to appoint a sub-committee consisting of the Rev. W. Moore Ede, Dr. Rutherford, and Dr. Henry E. Armstrong, to inquire into the expenditure on food in the Newcastle Infirmary, and to compare it with that of other institutions of a similar character.

EXPENDITURE ON NURSES.

One portion of the evidence given by the secretary of the Sunderland Infirmary related to the expenditure on nurses and servants, which, at Sunderland, amounts to £6 4*s.* 5*d.* per occupied bed, while the cost at Newcastle is £7 7*s.* 4*d.* Mr. Robinson explained that the chief reason why their expenditure on this head was so low was in consequence of their nurses being provided by the Tottenham Deaconess Institution, which sent their nurses thoroughly trained and qualified to do all the work required; they, in return, contributing only £300 per annum to that institution. The same plan could not be adopted at Newcastle, because it is a rule with the Tottenham Institution not to send nurses where there are medical students.

The committee are of opinion that, as we cannot have the benefit of the nurses from the above institution, the expenditure on the nursing department cannot well be reduced to any appreciable extent.

EXPERIENCE OF THE FREE HOSPITAL IN SUNDERLAND.

As the system of admission by letter has been often called in question, the committee made a lengthy inquiry into the experience of the Sunderland Infirmary, in which, prior to 1877, letters of admission had been used, and which since that year has been conducted as a free hospital.

Mr. Robinson the secretary of the Sunderland Infirmary, stated as follows :—

For many years before 1877 it was felt that the admission of in-patients to the Sunderland Infirmary by subscribers' tickets was attended by many disadvantages.

1. Acute cases were often deterred from applying by the difficulty of obtaining tickets.

2. Many cases applied furnished with tickets which were undesirable cases for admission.

3. The subscribers' ticket never covered the cost of the patients' stay in the house, while, at the same time, the subscriber was left under the impression that his subscription was ample to do so.

4. The system of buying the right to have a patient admitted was thought to be wrong in principle.

5. Very many small subscriptions, such as those from workmen, were withheld because of inequality.

It was also found that the free out-door Dispensary was greatly abused, and two principles were ascertained, namely: That in-door hospital relief should be untrammeled and free; that out-door dispensary relief should be administered on the provident system; and in 1877 this double change was effected.

The subscribers to the Infirmary were asked to give up their privilege of recommending in-door patients. They were given one ticket for every £1 1*s.*, which entitled a patient to treatment at the Provident Dispensary.

The Infirmary gave up the premises it had occupied as a dispensary to the New Provident Dispensary, and made it a grant of £500 a year in consideration of the patients recommended by its tickets being treated there.

Mr. Robinson further said that several persons had withdrawn their subscriptions in consequence of the change, but that most of them had since become subscribers, and that the Infirmary had not been in debt since the free system had been introduced; because the workmen's payments far more than counterbalanced any loss of subscriptions. He further informed the committee that since the hospital had become a free institution, the workmen have adopted a penny per week plan in over 200 works and firms in Sunderland. This money is collected at the offices, with the exception of two or three cases, where the workmen collect it themselves.

Mr. Robinson also informed the committee that, in addition to the regular Hospital Sunday collections, they had lately introduced the practice of setting apart the first Sunday in May as Hospital Sunday for Sunday Schools, the contributions received in this way being devoted especially to the children's wards.

He also reported that the removal of the out-door department, and the making the dispensary to a very great extent a provident institution, had not occasioned hardship to the poor, and said: "As far as I am able to ascertain, there is no hardship inflicted upon the poor by the present arrangement; the working classes are perfectly satisfied; and from inquiries I have made among the men, and from other sources, no complaints are heard of."

He also informed the committee that the number of out-patients and casual patients treated at all the medical charities in Sunderland last year was 4,480.

In Newcastle the number was as shown in following table:

*Number of Out-patients and Casuals treated in 1885 at the Medical Charities of Newcastle and Gateshead.*

| | |
|---|---|
| Infirmary, out-patients | 4,932 |
| Infirmary, casuals | 27,811 |
| Newcastle Dispensary, letter patients | 7,577 |
| Newcastle Dispensary, casuals | 13,426 |
| Gateshead Dispensary, letter patients | 3,133 |
| Gateshead Dispensary, casuals | 9,841 |
| Children's Hospital | 2,483 |
| Eye Infirmary | 5,000 |
| Hospital for Diseases of the Throat | 892 |
| Hospital for Diseases of the Chest | 538 |
| Hospital for Poor Women | 131 |
| Lying-in Hospital | 268 |
| Lying-in Charity, Gateshead | 109 |
| Total | 76,141 |

THE INFIRMARY AS A FREE HOSPITAL.

The committee are of opinion that the time has come for abandoning the letter system, and making the Infirmary a free institution. In making this recommendation they have the unanimous support of the medical board.

As this recommendation is one of great importance, and one on which difference of opinion exists, they print a letter received from Mr. N. G. Clayton, in which the chief arguments against the proposed changes are forcibly stated:—

Newcastle-upon-Tyne, 17th Nov., 1886.

Dear Sir,—As the question of making the Infirmary a free hospital may very probably come up for discussion when I am not present, I would ask the permission of the chairman to allow this letter to be read as an expression of the opinion of one who has long been connected with the Infirmary.

There is no magic in the word "free" as applied to a hospital. It simply means that the admission by letter shall be abolished.

At present the Infirmary is a "free hospital" so far as regards cases of accident and emergency; and the casual department, relieving, I think, 27,000 patients in the year, was "free" till the other day, when a charge of 3*d.* per patient was, very properly, as I think, put on. My reasons for wishing to retain the present system as regards in-patients are briefly these :—

1. The Infirmary was originally established for the "Sick and Lame Poor of the Counties of Northumberland, Durham, and Newcastle-upon-Tyne," and it it still retains its character in this respect. Severe and difficult cases are sent from all parts of the two counties in consequence of the excellence of the medical staff, and the other advantages of the hospital. A glance at the subscription list will show that a large proportion of the permanent subscriptions come from residents in all parts of the counties of Northumberland and Durham, and I should fear that the making the Infirmary a free hospital would curtail this source of supply, as it would then be looked upon as a local hospital for Newcastle and Gateshead and their suburbs only.

2. It would be unjust to the county subscribers and patients. At present, if a country practitioner has a case which baffles him, or where he thinks that the medical or surgical treatment and nursing of the Infirmary would be beneficial, he, or his patient, gets a letter from a neighbouring subscriber, and the patient, armed with this letter, goes on a Thursday, and if there is room, and the case is pronounced suitable by the medical staff, he is admitted. If the hospital were free, it would be a case of "first come first served," and the country patient would be somewhat in the position of the cripple at the pool of Siloam. He might incur the expense and risk of a journey or two, and not get in after all. The matter would be entirely at the discretion of the resident staff, and the county subscriber would have to ask as a favour what he now has as a right.

I am at a loss to see what countervailing advantage would be gained. Under the present system cases of emergency are amply provided for, and the benefits of the hospitals are certainly not lost through want of letters of admission, as it is always more or less full, and, sometimes crammed. It is very little hardship on town patients to seek for a letter when their cases are not pronounced to be pressing. Where a hospital is in the midst of a poor and dense population, as in Birmingham or the East End of London, I can understand that letters of admission may be dispensed with, but the circumstances of a hospital having, in addition, the character of a county hospital, and drawing patients from a large area, are entirely different. The evidence of the house governor shows that the privilege of giving letters is not abused, and the permanent staff of the Infirmary have the right of refusing unsuitable cases and returning the letters to the donors.

Under these circumstances, and with the present financial condition of the Infirmary, I hope that the committee will pause before they recommend a change which may curtail the income of the hospital, but certainly will not increase it.

I may add that, if the working men, by a systematic levy, increase their subscriptions materially, additional letters could, and no doubt would, be sent to the different works in return.

Yours truly,

J. H. Rutherford, Esq. N. G. CLAYTON.

The committee give the chief reasons why they, notwithstanding the objections raised, make this recommendation :—

1. The experience of Sunderland encourages them to believe that few, if any, of the present subscribers would withdraw their support.

2. From the evidence given, it appears that even now the cases admitted without letters are, on the whole, more suitable medically and less able to pay for medical treatment than those admitted by letter.

3. No injustice would be inflicted on persons living in the country, if medically fit for treatment in the Infirmary, for such cases would be received as readily as those from the immediate neighbourhood.

4. Undoubted hardship is inflicted on the suffering poor by their being compelled to go in search of letters. Compelling them to go begging for a letter is injurious to self-respect.

5. The committee do not see how they can appeal to the working men of the district to contribute 1*d*. per week, if, when they have so contributed, they are compelled to go in search of a letter before they can obtain admission. It does not follow that, because they have been contributing in connection with a certain firm, that the firm will always have a supply of letters for all the cases which may in any given year be required by their employés.

6. The committee believe that, even if some subscribers withdraw their subscriptions because they do not receive a *quid pro quo* in the form of letters, any such loss would be far more than counterbalanced by the increase in the contributions of the working men, which, they feel assured, would take place were the letter system abolished.

In support of the suggestions of the Food Committee that a more careful and thorough system be adopted with regard to the diet cards, we give a summary of the evidence given to the committee on this head :—

### THE DIET CARDS.

The diet cards are placed at the head of each in-patients' bed. On these cards the house physician or house surgeons enter the diet prescribed by the honorary physicians or surgeons. The nurse takes the cards, and the head nurse makes the summary. This is taken to the matron, who enters the particulars daily in the diet book, and gives the necessary orders to the housekeeper as to the quantity to be served out for preparation by the cook. The diet book is made up by the matron once a week, on the Friday afternoon, directly from the diet cards. During the rest of the week the matron is dependent entirely upon the reports of the nurses. Sometimes she found a discrepancy, but in her view it never amounted to much.

### DISCREPANCY BETWEEN DIET BOOK AND DIET CARDS.

That, however, was not the opinion of the house physician and surgeons, who were under the impression that, on some occasions, more food went up to the wards than was ordered, and that impression was, upon the whole, confirmed by a table presented by the house physician, showing considerable discrepancies between the diet books and cards of the 28th October of this year, and especially in regard to milk, as shown in tables submitted by the house surgeons. The house governor had called the attention of the house physician to the fact that the consumption of milk had risen from 1·3 to 1·6 pints per patient per day. A card was shown of a patient who had been in the hospital for three days, but as yet no diet was entered upon it. A test case was also submitted. A card, with the diet entry, "three pints of milk," on the interpretation of which the head nurses were by no means agreed, and the house physician cited an instance of gastric ulcer in which, with such a card, rice was also given.

### RECOMMENDATIONS.

Having regard to the importance of diet in the cure of disease, and in promoting recovery, your committee are of opinion (1) that the diet tables should be carefully revised; (2) that the diet prescribed should be entered on each patient's card without delay, on his admission; (3) that the nurses should be instructed on sick diet; (4) and especially to render with accuracy the prescriptions of diet on the cards; (5) that the matron or superintendent of nurses should be held responsible for the correspondence day by day between the diet as prescribed by the medical staff on the cards and as entered in the diet book, and served to the wards; and (6) that the house governor should occasionally compare the diet cards with the diet books, and report to the House Committee any discrepancies he may find.

### NURSING DEPARTMENT.

This department is under the superintendence of the matron who controls and is responsible for all the female servants in the Infirmary, including the housekeeper and cook. There are at present 41 nurses, with an average of 270 occupied beds, or one nurse to every six and a half occupied beds. At Sunderland Infirmary there is one nurse to every six and a quarter occupied beds, slightly less than Newcastle, and there are no students to take part in dressing and attending to the patients. Therefore, the contention that the students contribute largely to the Infirmary by the value of the services they render does not find much support. The existing provision made for the instruction of the nurses in cookery for the sick, or in the ordinary duties of their office, seems to be inadequate.

### CLASSES FOR THE INSTRUCTION OF NURSES.

The nfirmary cannot be an efficient training school of nursing without thorough and systematic instruction, and your committee recommend that this important department be placed under the superintendence and care of an honorary medical officer, who shall be responsible to the House Committee.

### MEDICAL EXPENDITURE, ANTISEPTICS, ETC.

The committee inquired into the increase of the expenditure connected with the antiseptic treatment, and found that it was accounted for, in part, by the change in the classification of the accounts; and, in part, by the increase in the number of operations. In connection with this head, it was pointed out that the death-rate on operations was 15·6 in

1874; and that since the antiseptic treatment had been introduced it had fallen to an average, for the ten years, of 3·7, and last year was only 2·3 per cent.

The expenditure for surgical appliances, antiseptic dressings, drugs, etc., in the Newcastle Infirmary during the year 1885 was £2,730 8*s.* 3*d.*, having advanced almost by leaps and bounds from £786 4*s.* 11*d.* in 1875. This rapid increase of expenditure depends principally on the great number and severity of the surgical cases requiring antiseptic treatment. But it compares unfavourably with Edinburgh, where many of the surgical cases are also very severe. In 1884, with 599 as the average daily number of in-patients in the Edinburgh Infirmary, the cost for medicines, bandages, antiseptic dressings, surgical and other instruments, and liquors was £3,682 1*s.* 3*d.*, or £6 2*s.* 10*d.* per occupied bed. At Newcastle, with an average of 219·5 in-patients, the cost during 1884 under the same head was (£2,012 18*s.* 5*d.* + £358 0*s.* 9*d.*) £2,370 19*s.* 2*d.*, or £10 12*s.* 9*d.* per occupied bed for the year. In the accounts of the Royal Infirmary, Edinburgh, wines, spirits, and malt liquors are classified under the head of medical expenditure, the total amount of which for 1885 was £3,749 2*s.* for a daily average of 595 in-patients, or £6 5*s.* 0¾*d.* per occupied bed per annum; while in Newcastle Infirmary it was £415 3*s.* 5*d.* for stimulants, £2,730 8*s.* 3*d.* for medicine, and making a total of £3,145 11*s.* 8*d.* for a daily average of 234·7 in-patients, or £13 7*s.* 7¾*d.* per occupied bed per annum. Had the Edinburgh rate of medical expenditure not been exceeded in Newcastle Infirmary there would have been a saving on the year 1855 of £1,664.

Professor Annandale, in a communication addressed to Dr. Bruce, states that, though the treatment in the Royal Infirmary, Edinburgh, has been much modified, the antiseptic dressings do not cost much less. With the view, however, of checking any unnecessary extravagance, each of the surgeons of that institution is furnished monthly with a form, showing the quantity and cost of the surgical dressings issued from the stores to his ward during the preceding month.

In Newcastle Infirmary there is no check of this kind. Tne whole matter ls in the hands of the Medical Board, but the sub-committee have once in every three months "to call for the report of the sub-committee of the Medical Board on the subject of the drugs, medicines, and instruments and apparatus of the institution." This, however, does not amount to an audit, and does not invest the House Committee with power involving responsibility for the medical expenditure.

RECOMMENDATIONS.

Your committee recommend, (1) that this expenditure be placed under the direct control of the House Committee; (2) that all orders for medical and surgical stores and appliances be signed by the house governor, under the sanction of the house committee on the usual forms; (3) that all such orders be issued on the requisition of the Medical Board, or a representative of that Board; (4) that a separate account be kept of the expenditure of each honorary surgeon and assistant surgeon, and that as early as practicable in each month, on forms similar to those used in Edinburgh Royal Infirmary, a return be made to each surgeon and assistant surgeon of the expenditure on his patient for the month immediately preceding; (5) that no goods be received without an invoice, which must be compared with contract prices; (6) that the words "other than medical" be struck out of Rule 3, p. 17, and that the house governor have charge of the medical and surgical as well as the general stores; (7) that no surgical instrument or appliance exceeding the value of £5 shall be ordered without the formal sanction of the committee; and (8) that the requisitions from the Medical Board from time to time, be presented to the House Committee, and if approved, signed by one of the members appointed to that duty.

THE INFIRMARY AS A SCHOOL OF PRACTICAL MEDICINE AND SURGERY.

The Infirmary has long been a school of practical medicine and surgery, and, though humble in its beginnings, has been not the least important factor in the success of the College of Medicine. The number of students entered at the hospital for practice at the present time is 115—all of them from the College of Medicine. Of these 38 were full students, entered to take their complete course at the college, and about 40 new students from the metropolitan hospitals, or 78 new entries in all. The perpetual fee for hospital practice is twenty-five guineas; for one year, twelve guineas; for six months, eight guineas; and for three months, five guineas. These fees cover clinical instruction. At present they amount to something like £1,000 per annum, and are at the disposal of the honorary physicians and surgeons, and are collected by them. From £70 to £80 a year is spent upon the library, which is open to governors and to the infirmary staff free, and to other practitioners and students on becoming subscribers to the library fund of ten shillings annually. The Museum, though remaining the property of the Infirmary, has been removed to the College of Medicine, and is not now supported out of the fund received from students for hospital practice. The balance is entirely in the hands of the Medical Board; the House Committee have no control over it whatever, and its amount and distribution are not even reported to them. These arrangements, though not specifically mentioned in the statutes and rules adopted by the governors of the Infirmary in 1883, are not inconsistent with them, and are in accordance with the general practice of English hospitals. Your committee, however, cannot but think that it would harmonise better with the purely honorary position of the staff, whose services never can be paid in gold, and with the spirit and genius of the institution as a medical charity, that the fees received by them should be solely and purely for clinical teaching. Your committee are of opinion that, without interfering with the action of the medical staff in charging fees for such instruction, a charge of £5 5s. per annum should be made to every student for the use of the Infirmary, to be paid directly to the treasurer of the Infirmary, and acknowledged by him.

In such a course the Newcastle Infirmary would only be following the example of the Royal Infirmary of Edinburgh, where every student is required to pay a fee for hospital practice, apart from clinical instruction, for which he receives a ticket from the treasurer. The cost of a perpetual ticket is twelve guineas, the total amount accruing to the funds of that institution from students for tickets of admission in 1885 being £4,083 19*s.* The proposed charge for the privilege of using the Infirmary would, at a severe financial crisis like the present, secure the welcome addition of something like £500 per annum to the funds of the Infirmary; and the separation of the fees for clinical instruction from the fees for hospital attendance, with the hearty approval of the Medical Board, would go far to awaken the popular enthusiasm, on which we rely, to start the noble old institution on a new career of prosperity and usefulness.

THE INDOOR STAFF ARRANGEMENTS.

Your committee also submit whether it would not contribute to the quiet and decorum of the institution, as well as to its efficiency as a training school for medical students and nurses, to assimilate its arrangements to those of the Royal Infirmary, Edinburgh, and to provide board and lodgings only for the house physicians and surgeons, whose numbers should be regulated by the number of occupied beds, and whose tenure of office should be defined.

THE HONORARY STAFF.

Your committee further submit to the Court of Governors whether the appointment to a position on the honorary staff should not, as in Edinburgh, be limited to a term of seven years.

PROVISIONS FOR EXTENSION.

Your committee are satisfied that the adoption of the antiseptic treatment renders the erection of a new hospital no longer an urgent sanitary question; yet it is worthy of consideration by the governors whether any fresh extension which may in future years be required, should not be provided for, in consultation with the authorities of the Medical College, and form the nucleus of a new hospital, to be erected in a position near the greater portion of patients, and convenient to the staff and the students, with every advantage of air, light, and quiet, and whether it would not be the truest economy early to anticipate that future.

DUAL GOVERNMENT.

The committee are of opinion that the present method of dual government by the House Committee and the Medical Board is not conducive to the interests of the Infirmary, and is the cause of much needless friction, and a divided responsibility. They recommend that in future there be only one governing body, and that on this body there should be a fair representation of the medical staff.

The committee further recommend a revision of the method of election of the House Committee. Provision should be made for working men representatives from factories which contribute to the Infirmary being on the electoral body, and measures should be taken to secure a larger proportion of workmen's representatives on the committee.

ALTERATION IN THE METHOD OF ELECTING THE HONORARY STAFF.

The committee desire to call attention to the very unsatisfactory method under which the election of the honorary medical staff is conducted. On the skill of these gentlemen the reputation and efficiency of the Infirmary, as a medical institution, depends, yet their election is dependent on their skill as canvassers, or the number of personal friends who canvass for them. This must always be the case when success depends on securing a majority of the votes of a large body of subscribers, who know little of the medical fitness of the applicants. The committee, therefore, recommend that, in future, all elections to vacant posts at the Infirmary be placed in the hands of a committee of selection. Such committee might be the House Committee, reorganised as suggested above, or a special committee appointed for that purpose.

These changes, which your committee consider of great importance, would require sundry alterations in the wording of the present rules.

OUT-PATIENTS AND CASUALS.

Out-patients may be divided into two classes: (*a*) Those whose cure is not completed when they leave the Infirmary and who continue to attend the Institution in order to receive further medical supervision and treatment till their cure is, as far as possible, effected; (*b*) Those whose disease or injury is such as not to require admission into the Infirmary, but who need medical advice, medicine, or instruments. These receive attention from the medical officers of the Infirmary at the Institution. These patients receive attention either on account of out-patients' letters which they bring, or because coming as casuals, their diseases are such as require more or less prolonged medical treatment.

Casuals consist of those who visit the Infirmary for medical advice or treatment without any letter of recommendation or any other certificate of fitness, either medical or as regards their pecuniary circumstances.

The number of out-patients in 1885 was 4,392; of casuals, 27,811. The out-patients have more than doubled, the casuals nearly trebled, in the last ten years. A charge of 3*d*. was imposed in August last, and since then the number of casuals has fallen considerably. The cost of the out-patient and casual department for 1885 is estimated at £1,440. This estimate has been challenged, but the committee are of opinion that it is fairly accurate. Our inquiring into the class of cases treated in the casual department, the committee were startled on being informed by Mr. Waldy, who has much experience among the casuals, that he believed rather more than one-half of the males, and less than half of the females, were cases of persons suffering from venereal disease.

INVESTIGATION OF CASES.

The committee ordered a record of the cases to be made for the following week, December 14th to 20th, when, out of a total of 161 cases, 41 were reported as venereal—*i.e.*, one-third, and not one-half, as stated. It should, however, be mentioned that the committee had ordered that the names and addresses of all applicants should be taken, in order that inquiries should be made into their pecuniary circumstances, in order that they might discover how far the charity was abused. Many refused to give the required information, and left without medical advice rather than give their addresses. The number of casuals for that week was one-third below the average. The committee sent the names and addresses which were obtained to the Charity Organisation Society, who very kindly undertook the investigation of the cases.

From the report of the Charity Organisation Officer, it appears that the first 33 cases investigated give the following result:—five were in receipt of two pounds and upwards per week, two had given false addresses, five were in receipt of parish relief, and the remaining twenty-one were persons whose circumstances may be said to have justified their seeking medical assistance, though some could afford to make a small weekly payment to a club or provident dispensary if there were one.

EXCLUSION OF VENEREAL CASES.

The committee are unanimously of the opinion that no male venereal cases ought to receive treatment at the expense of the institution. It is an abuse of the charity never contemplated by the subscribers. The male lock ward was closed during the year 1885, and only a few cases of venereal disease have since been admitted as in-patients.

The committee most earnestly hope that a stringent rule will be made excluding all such cases from the Infirmary, and that the other medical charities which undertake the work of supplying medical advice and medicine will refuse to permit their funds to be expended on the treatment of this class of case.

INDISCRIMINATE MEDICAL CHARITY.

The mere fact that, in something like 75,000 cases, gratuitous medical relief has been given in Newcastle and Gateshead during the year 1885, reveals the pressing need of reform. It is admitted on all hands that such cases should be sifted; but by what method, and who is to apply it? The increase of the out-patients in one year of nearly a thousand, from 3,947 in 1884 to 4,932 in 1885; and of casuals, from 23,513 in 1884 to 27,811 in 1885, is not only startling, but, if it represents real disease, appalling. Your committee make every allowance for the distress and poverty occasioned by the depression of trade; but Sunderland has suffered as much, if not more than Newcastle, from depression of trade, and yet only 4,480 received gratuitous medical assistance as out-patients or casuals. Large numbers of the working classes are members of the Provident Dispensary, and as far as we can ascertain, not only is no undue hardship inflicted on the poorer section of the population, by this curtailment of out-patient relief, but the working classes are themselves thoroughly satisfied with the existing arrangements. Indiscriminate medical charity is not only injurious to the poor themselves, by weakening their sense of independence, and encouraging improvidence and unthrift, but tends probably to create and develop the physical ailments which it is meant to heal. There are morbid conditions dependent in some persons on the continuity of the mental impression that something ails them, and whatever strengthens that impression is likely to counteract the benefit of medical treatment. For such and similar cases the atmosphere and the concomitants of the out-patient room cannot be considered wholly favourable. To start a new train of thought or feeling is the surest way to break the power of the old unhealthy habit, and to touch their pockets is for many the shortest way to the brain. To think they are better is sometimes to be better. But what people do not pay for they are apt not to appreciate, and are not so likely to benefit by. In a time of depression decent people with long and chronic complaints may, more or less, be excused for seeking the aid of a charitable institution, but people with any means need to be reminded that, after all, it is really a very wasteful way of getting medical attention. The time spent, the exposure to cold, the risk of infection, the absence of selection, and the danger of contact may be too high a price to pay for gratuitous medical relief. What is wanted is the thorough, if not severe investigation of the fitness of cases at the hospital itself. The difficulty is one of all our charitable medical institutions, and it can only be surmounted by insisting upon the name, the address, occupation, and employer of every person seeking relief, being properly entered in the books, and by the hearty co-operation of the organisations for charitable and parochial relief.

ABOLITION OF OUT-PATIENTS AND CASUALS.

The committee are of opinion that the whole of the casual department should be abolished, and out-patients restricted to those who have been inmates of the Infirmary, and who, though well enough to leave the institution, leave before their cure has been completed. This would effect a saving of something like £1,000 a year to the institution.

The committee do not, however, make this recommendation so much on grounds of economy as on the abuses which we find exist, and the overlapping of the medical charities which is occasioned.

The Infirmary exists for the hospital treatment of persons who cannot be adequately treated in their own homes. The Dispensaries of Newcastle and Gateshead are maintained for the treatment of those who do not require hospital treatment, and yet are too poor to pay for medical advice. No hardship would be inflicted on the poor by the abolition of the out-patient and casual department of the Infirmary, for those who have hitherto come to the Infirmary, and are neither venereal cases nor in a position to pay for treatment, will be eligible for assistance from the Dispensary.

The most serious objection to the proposal is on the ground that the medical students will lose the experience they acquire from attending to the casual patients, and that those who come as casuals are often found to be persons who need hospital treatment.

There ought not, however, to be much difficulty in arranging for the students seeing the casuals at the Dispensary, nor in arranging that cases which, when they present themselves at that institution, are found to require hospital treatment, should be sent to the Infirmary. In truth, the casual and out-patients' rooms are not the places to study disease till the student has become thoroughly familiar with its phenomena, as seen in the wards of the hospital.

THE INFIRMARY, AS COMPARED WITH OTHER HOSPITALS.

The committee desire to put on record their sense of the great value of the services of the House Governor, who has, throughout this lengthy inquiry, assisted them with valuable information, and the excellence of whose management is attested by the fact that, notwithstanding the large expenditure on food and drugs, over which he has no control, the total cost per bed of patients in the Newcastle Infirmary, is among the lowest of any provincial hospital in England, as may be seen by reference to the following table of comparison made with twelve of the leading hospitals chosen at random :—

*Comparative cost per Occupied Bed in the Year* 1885, *in Twelve large General Hospitals in Industrial Centres*:—

| Occupied Beds. | Name of Place, etc. | Cost per Bed. £. | s. | d. |
|---|---|---|---|---|
| 237 | Birmingham General | 56 | 6 | 0 |
| 113·6 | Bradford ... about | 53 | 0 | 0 |
| 595 | Edinburgh, Royal | 56 | 5 | 9 |
| 368 | Glasgow, Western | 50 | 18 | 0 |
| 96 | Hull | 65 | 16 | 4 |
| 253 | Leeds | 56 | 7 | 9 |
| 161 | Liverpool, Southern | 49 | 0 | 5 |
| 620 | London Hospital | 68 | 5 | 10 |
| 253 | Manchester, Royal | 60 | 1 | 0 |
| 138 | Nottingham | 55 | 3 | 8 |
| 128 | Sunderland (12 months of 1885) | 42 | 3 | 9 |
| Say 200 | Wolverhampton | 52 | 5 | 2 |
| | | £665 | 13 | 8 |
| 255 | Average. Average of twelve | £55 | 9 | 5 |
| 235 | Newcastle Infirmary | £50 | 14 | 9 |
| | Do. for first three quarters of 1886, at rate of | £48 | 15 | 3 |

The committee, in bringing this report of their investigation into the expenditure of the Infirmary to a close, desire it to be distinctly understood that, though they have made many recommendations as to various points of re-organisation, they are of opinion that, on the whole, the Infirmary has been well managed according to the rules and regulations which have been in force, and that there has been no wilful waste or wanton extravagance. It is greatly to the credit of all the officials of the Infirmary that such a large institution, subjected to so searching and thorough an investigation as that which this committee has conducted, should have so well stood the test.

The officials have carried out the existing system well, but it is the opinion of the committee that the system hitherto in force is, as they have indicated, defective in many respects.

GENERAL SUMMARY.

For convenience of reference, we here give a summary of the recommendations of the Committee of Inquiry :—

1. Removal of children to the Children's Hospital.
2. Opening of wards for patients who contribute to their own maintenance.
3. Abolition of admission letters.
4. Revision of dietary and reduction on food expenditure.
5. Reduction on stimulants.
6. Improved system of keeping and checking diet cards.
7. Systematic instruction and training of nurses.
8. Adoption of the Edinburgh method of keeping accounts of dressings, drugs, etc., so as to show the expenditure on the patients of each member of the medical staff separately.

Abolition of casuals and limitation of the out-patient department.

Payment of £5 5s. per annum by all medical students.

Amalgamation of Medical Board and House Committee.

Revision of system of election of the House Committee.

Even should experience prove that the committee's expectation as to the possible reduction in expenditure be realised, there would still continue to be an excess of expenditure over income of several thousand pounds per annum. The committee have endeavoured, therefore, to ascertain whether there is any source from which additional income can be obtained.

A number of medical institutions have grown up of late years, all of which appeal to the public for support, and among these various medical charities there has, hitherto, been a want of system and due co-ordination. Of the sum raised by the Hospital Sunday and Saturday Fund, only one-half goes towards the support of the Infirmary, the other half being divided amongst the numerous smaller medical charities.

WORKMEN'S CONTRIBUTIONS.

Nothing will tend so much to stimulate the benevolence of the general public, and to increase the support of the employers of labour, as the enlarged and systematic contributions of the working men themselves to the funds of the Infirmary. It is their institution. It exists for them, their wives, and families. Its doors are open night and day to all cases of accident, injury, and emergency, without letter, and without demur. Its appliances and skill are at their disposal in their hour of agony. They have only to think of the multitude of valuable lives it has saved, of the greater multitude of sufferers it has restored to health, to feel that a small contribution once a year cannot adequately express their appreciation of an institution to which, as a class, they owe so much. The Hospital Saturday Fund amounted in 1885 to £1,507 8*s*.4*d*., and in 1886 to £1,770 7*s*. 1*d*. A certain proportion of the Hospital Sunday collections must also be placed to the credit of the working classes. But 1*d*. per week from every workmen at Elswick Works alone would considerably exceed that amount. And the general adoption of systematic weekly giving to the funds of the Infirmary, in the workshops on the Tyne, by employers and employed, is not more than the necessities of the case require, and less than that would leave its energies greatly crippled. Nor will the working men fail to notice the danger to the future prosperity of the Infirmary arising from the diversion of funds into the coffers of other institutions, which, whatever their merits, are not capable of grappling with the injuries and the diseases incident to the industries of the district. Working men recognise, that the Infirmary is a genuine public hospital; that, while it has done considerable service to medical education, it has not been set up by specialists for their private advantage; and that its future will largely depend upon the support given by the working classes. It is on these broad grounds that your committee feel assured that an appeal to the sympathy and help of the working people will not be made in vain, and the full and adequate representation of the industrial establishments of Northumberland in the Court of Contributors and Governors will not be the least important guarantee that in the future the highest economy compatible with efficient administration will be attained.

The committee are strongly of opinion that one of the most important needs of the present time is the introduction of a more clearly defined division of labour between the medical charities of the town, so that each may have its special function, and thus there may be less overlapping and waste of money and energy; and they venture to commend this question to the consideration of the Committee of the Hospital Sunday Fund, who, being in communication with all the medical charities, have perhaps the best opportunities of getting this much-needed reform carried into effect.

Printed by WHITING & Co., 30 and 32, Sardinia Street, in the Parish of St. Giles's-in-the-Fields, in the County of Middlesex, and Published by WILLIAM KEEP, at the same address, in the said Parish and County, as a Special Supplement to THE HOSPITAL of February 5th, 1887.

